# Streaming ambivalence: Livestreaming and indie game development

**DOI:** 10.1177/13548565211027809

**Published:** 2021-07-28

**Authors:** Felan Parker, Matthew E Perks

**Affiliations:** St. Michael's College, University of Toronto, Canada; University of Waterloo, Canada

**Keywords:** Cultural production, game development, game studies, independent games, indie games, live streaming, platforms, Twitch

## Abstract

Commercial game makers at all scales of production have increasingly come to incorporate livestreaming into every stage of the game development cycle. Mainstream hits like *Fortnite* and *League of Legends* owe their ongoing success in no small part to their massive uptake by streamers, and triple-A releases from major publishers can reliably expect significant attention on streaming platforms. But what about smaller, lower budget games? For independent game developers, the costs and benefits of streaming are less clear. Based on interviews with small commercial indie developers in Toronto and Montréal, this article critically examines different discourses around streaming and commercial indie games, focusing on developer perceptions of the benefits and risks of streaming and its impacts on indie game-making practices, including production, promotion, and community-building. Contrary to persistent popular myths about streaming as the key to ‘discoverability’, commercial indie game development remains a precarious form of cultural work, and indie games collectively attract only a tiny fraction of the overall audience on streaming platforms. There is a high level of uncertainty about the factors that led to a given game’s success, leaving many indie developers ambivalent about leveraging influencer attention and even as they commit significant time and energy trying to doing so.

## Introduction

Without question, livestreaming is changing the industry and culture of digital games. Twitch in particular has been built up as *the* platform for users to broadcast themselves performing play and vie for the elusive social and economic rewards of online celebrity. Viewers consume billions of hours on a monthly basis, interacting with streamers and fellow spectators via live chat in ways that spill onto social media and ripple outward to shape popular tastes, modes of communication, cultural attitudes, and dominant play styles in game culture (Taylor, 2018). Twitch and competing platforms like YouTube (and their parent corporations Amazon and Google) extract massive profits from all this engagement via advertising, sponsorship deals, and various fees, guiding user attention to specific channels via front page ranking and recommendation algorithms (Partin, 2019). Commercial game makers at all scales of production have increasingly come to incorporate streaming into every stage of the game development cycle. Mainstream hits like *Fortnite* and *League of Legends* owe their ongoing status as bona fide pop cultural phenomena in no small part to their massive uptake by celebrity and amateur streamers alike, and triple-A releases from major publishers can reliably expect significant attention on streaming platforms, in some cases achieved by paying streamers directly to play (Lanier, 2019). But what about smaller, lower budget games? For independent game developers, the costs and benefits of streaming are less clear.

Indie developers are acutely aware of the centrality of streaming in the contemporary game industry ecosystem, but they lack the resources, brand recognition, and dedicated marketing teams of big-budget giants. There is a persistent popular myth that streaming and related forms of online content creation are a golden key to indie game ‘discoverability’ and ultimately sales, and that Twitch streamers, YouTubers, and other game-based content creators and influencers are the new gatekeepers of indie success (Phillips, 2018; Takahashi, 2016). However, commercial indie game development remains an extremely precarious form of cultural work (Whitson et al., 2018). A great diversity of game and non-game content is broadcast but popular blockbusters continue to dominate streaming platforms, attracting the highest profile celebrity-influencers and their legions of fans, as well as countless smaller streamers. With the rare exception of breakout indie hits like *Among Us* (Fenlon, 2020), indie games collectively make up only a tiny fraction of the overall audience. There remains a high level of uncertainty about the factors that lead to a given game’s success, leaving many indie developers ambivalent about leveraging influencer attention for sales even as they commit significant time and energy trying to doing so. Are streamers the golden key to success, a necessary cost of doing business as an indie, or platform capitalist snake oil? This article critically examines different discourses around streaming and commercial indie games, beginning with an overview of popular success stories, then focusing on developer perceptions of the benefits and risks of streaming and its impacts on indie game-making practices, including production, promotion, and community-building.

The body of academic research on Twitch and game streaming continues to grow, and scholars have investigated streaming services as platforms, the experiences of streamers marginalized on the basis of race, gender, sexuality, and mental health, the diverse forms of visible and invisible labor involved in streaming, cultures of game spectatorship, the possibilities of streaming for game development education, and the intersection of streaming and competitive esports (Consalvo and Phelps, 2021; Gray, 2017; Johnson and Woodcock, 2019a, 2019b, 2019c; Ruberg, 2020; Ruberg and Lark, 2020; Ruberg et al., 2019; Taylor, 2018; Walker, 2014). These insights directly inform our approach here, and we hope to expand and nuance this body of work by directing attention to the experiences of game developers with streamers and streaming platforms, extending the project of indie game studies and game production studies (Ruffino, 2021; Sotamaa and Švelch, 2021). We likewise build on critical work on the political economy of digital platforms, online influencers, content creators, and micro-celebrity and media and cultural industries research more broadly (Abidin, 2018a; Bishop, 2020; Duffy, 2017; Duffy and Hund, 2015; Duguay, 2019; Nieborg and Poell, 2018). Ultimately we argue that, contrary to popular success stories, the impacts of streaming for indie game developers are complex and uncertain, and their ambivalence is characteristic of contemporary platformized cultural work.

Our findings are based on semi-structured interviews with 12 indie game developers based in Toronto and Montréal, Canada (see Table 1 below) selected using a combination of purposive sampling leveraging past connections and snowball sampling. Canada is the third largest producer of digital games internationally, and both cities are significant hubs, encompassing game-making activity from AAA to DIY. Almost 90% of Canadian studios, including all of our interviewees, fall into the category of ‘small’ or ‘micro’ operations with less than 25 employees (Nordicity, 2019). Our focus here is on commercial indie game developers who primarily make original, creator-owned games, usually distributed digitally, in a variety of production contexts.

**Table 1.** Study participants

Pseudonym	Studio size	Roles	Projects at time of interview
Hugh	18	Creative direction, writing	4 releases, 1 in development
Helena	9	Communications, community management	6 releases, several more as publisher for other studios
Melvin	6	Cofounder, game design, business development	1 released, in post-development
Tessa	6	Community management and quality assurance	2 released, in post-development
Holly	3	Studio director, operations lead	1 released, 1 in post-development
Lauren	N/A	Coworking space project manager	Worked with indies and streamers as coworking space staff
Stuart	3	Cofounder, game design	1 in development with studio, several independently released
Curtis	2	Founder, game design, art direction	5 released
Charlie	6	Coworking space cofounder, producer	6 released
Carolyn	3	Cofounder, art direction	1 released, 1 in development
Tom	1	Solo developer	3 released
Christopher	5	Communications	3 released, 1 in development

‘Developer’ here includes all kinds of game workers, not limited to studio leadership or traditional ‘creative’ roles, but also frequently overlooked roles in commercial game-making like marketing and community management (Perks, 2020). In some cases, due to the shifting nature of indie cultural work, developers are responsible for multiple areas, while others are in more dedicated roles. All participants are embedded to varying degrees in local and translocal indie scenes and most are personally acquainted via community organizations, coworking spaces, and social events, as well as larger global networks of indie developers (Parker and Jenson, 2017). In addition to individual experience, these interconnected communities of practice inform developer understanding of streaming through informal knowledge-sharing and formal initiatives, such as events for developers to meet local streamers organized at coworking hubs.

Interviews took place in single sessions in 2018 and 2019, usually in studio offices or coworking spaces, and participants were asked open-ended questions about their experiences with game livestreaming, how they interact with streamers, the impacts of streaming on various aspects of development, differences between streamers and other kinds of intermediaries like journalists, and the role of streaming platforms themselves. Interview data was transcribed then collaboratively coded and analyzed according to emergent themes, allowing us to synthesize on the ground stories, perspectives, and attitudes. Participants were given the opportunity to review the article and quotations before publication, and all names have been anonymized. This research is part of the larger Indie Interfaces project, and in addition to these interviews, our findings are informed by extensive interviews and ethnographic work conducted with indie game developers and cultural intermediaries between 2015 and 2019, during which time the potential importance of streaming for indies became increasingly apparent.

### Streaming success stories and cultural intermediation

To set the stage for the present research, it is important to consider the wider industry context and popular narratives around indie games and streaming. In the wake of digital distribution, cheap bundling of games, and increased interest in smaller games, commercial indie games are now widely understood to be an oversaturated market, making it difficult to stand out (Keogh, 2018). In light of these concerns (whether or not they are accurate), streaming appears to be an ‘implicit low-intensity marketing’ solution to the problem of discoverability (Kerr, 2016: 135). Popular streamers command the attention of hundreds, sometimes thousands of eyeballs, and if they are playing your game, then there is a presumed opportunity to convert them to customers and fans. Journalist Jason Schreier underscores the role of streamers and YouTubers in the success of two breakout indie hits, *Stardew Valley* and *Shovel Knight*. In his account, ‘early streams and videos generated more buzz for *Stardew Valley* than any press outlet’ (2017: 77), and ‘when huge YouTube channels like the Game Grumps later played through the [*Shovel Knight*] demo, they reached hundreds of thousands of people’ (2017: 180). These and other success stories about indie developers making it big thanks to positive attention from streamers and YouTubers circulate widely and inform game development practices. Like other indie success stories, these narratives tend to assume a linear path in which the passionate labor and creative vision of obscure independent creators, along with a little luck, translates into well-earned fame (Ruffino, 2013). The developers we spoke to frequently mentioned these and other examples, and a handful have found traction with streamers for their own games.

In many ways, game streamers resemble cultural intermediaries, those actors in a cultural field that connect cultural works to consumers (Matthews and Smith Maguire, 2014). Intermediaries such as community organizers, festival and showcase curators, critics, coworking space coordinators, and other behind-the-scenes actors are the connecting tissue that constitutes indie game culture as such (Parker et al., 2018; Perks et al., 2019). Aphra Kerr calls game streamers and online content creators

new cultural intermediaries who are taking the place of specialist game magazines and written game reviews. These players are generating advertising, sponsorship revenue and driving sales of games. They assist in the circulation, marketing and commodification of gameplay. (2016: 137); Mark R. Johnson and Jamie Woodcock go so far as to argue that streamers are making professional reviewers obsolete (2019)

Carolyn gestures to this as she tries to find the right word to describe what exactly streamers do for indie developers, suggesting ‘servers’, ‘advertisers’, and ‘sales people’ as possibilities, while Holly thinks of streamers as ‘tastemakers’ that draw attention to new games.

Our research suggests these accounts of influencers’ influence may be exaggerated. Certainly, game streamers can act as tastemakers in that they – at least sometimes – are able to expose consumers to previously unknown cultural products. But Kerr goes on to note that the paratextual content created by streamers ‘exists in an uneasy relationship’ to the game makers whose work they build their streaming careers on (Kerr, 2016: 137). This uneasy relationship is further complicated by the platforms themselves, who are themselves powerful intermediaries. For this reason, T.L. Taylor challenges reductive accounts of streaming as merely promotional, a framing that glosses over the more complex cultural-economic interdependences involved and the creative/cultural labor of streamers themselves (2018: 50–51). There is an important difference between ‘downstream’ intermediation of putting games in front of potential players associated with advertising and tastemaking, and ‘upstream’ intermediation between developers and powerful industry actors like publishers, platform-holders, and investors (Parker et al., 2018). This is further muddled by forms of ‘cross-stream’ intermediation between developers and journalists, curators, and community organizers whose ‘relational labour’ and networks of mutual support are far from obsolete and remain key to indie game development even if they do not directly engage consumers (Baym, 2015; Whitson et al., 2018). As we will show, streaming is not a simple or linear process of promoting cultural products to consumers, and in fact performs a wide variety of functions for a diverse range of actors to ‘transform private play into public entertainment’ (Taylor, 2018: 22), and indie game developers do not necessarily have much agency in this process.

Meritocratic success stories risk misrepresenting the work and complexities involved in both streaming and indie game development. In reality, only a small upper crust of indie games catch the attention of streamers and influencers in the first place, and the process by which they do so is anything but straightforward. These stories also ignore the ‘survivor bias’ of early adopters of new game production and distribution techniques; what begins as an exciting new ‘blue ocean’ quickly becomes a hyper-competitive ‘red ocean’ as other developers attempt to emulate the success stories (Mi, 2015) – indeed, breakout games like *Stardew Valley* and *Among Us* occupy significant platform real estate, making it that much more difficult for newcomers to capture attention. Melvin alludes to this, saying part of the challenge for developers is keeping abreast of new avenues for promotion and distribution, without falling into the trap of replicating strategies that no longer work. No doubt hard work, good ideas, and sheer luck play a role, but our research participants – including those that have found popularity with streamers – point to a more complex and ambivalent assemblage of actors, factors, and attitudes at play, suggesting that success stories are not the whole story.

### Streaming and indie game production

Unsurprisingly, the rise of streaming has influenced not only promotional strategies, but all aspects of game development, including the design process. Tom argues that ‘streaming games has changed the landscape of what kind of games are practical to build’, or at least what is commercially marketable. In the current moment, all game developers are compelled to keep the dynamics of streaming platforms in mind as they conceptualize, execute, iterate, and launch projects and support them post-release – even if they ultimately choose to ignore them.

#### Watching others play

The most subtle but important way that streaming shapes game design is that developers are able to covertly watch their games being played online. Watching streams and gameplay videos becomes an extension of playtesting for developers, which is particularly valuable for in-development games with public ‘early access’ releases, or completed games that may be continually patched, updated, and developed for months or years after release based on player reception, data analytics, platform changes, and other factors (Nieborg and Poell, 2018). This offers certain advantages compared to conventional private playtesting. Hugh compares it to watching ‘actual people’ playing at in-person exhibitions, but better. He is especially drawn to smaller streamers with low viewer counts, who he says are more likely to ‘play the game in a very similar environment to how they play the game if they were just playing without streaming it’. For Christopher, this removes the artifice of playtesting in the studio or at shows, because the players are playing without direct ‘coaching’ and scrutiny from the developers, resulting in something close to the ‘the real experience of a first time player’. This lack of scrutiny leads to less filtered, more actionable feedback according to Tessa, because streamers ‘don’t feel like [they] owe any amount of patience to the game to make you understand, which can come across as pretty harsh […] but at the same time, it’s fair’. Melvin remembers how watching streamers struggle with certain features of his game (which was not originally designed with streaming in mind) was revelatory, and helped identify key usability problems, bugs, and other issues to be fixed that were missed in regular playtesting. However, this also creates a new challenge for developers. As Hugh notes, if the game is too buggy or broken, streamers may bounce off of it, or viewers may decide, ‘well, there’s 7,000 other games released this year. I’m not buying this one’. If the developers aren’t able to make the necessary fixes promptly in response to issues flagged by streamers, says Christopher, ‘We’ve lost these players or these viewers’, the opposite of the desired effect. This illustrates the risk of unofficial playtesting in front of a live online audience compared to more controlled environments, as well as the ‘always-on’ grind necessitated by the shift to ongoing ‘games-as-a-service’ style development (Dubois and Weststar, 2021).

#### Designing for ‘streamability’

Different genres, styles, and features are considered more or less amenable to the performance of play, and most developers we spoke to considered ‘streamability’ and ‘watchability’ in the design of their projects from the beginning in hopes of increasing their platform ‘discoverability’ (Della Rocca, 2020; McKelvey and Hunt, 2019). Action-oriented, competitive, and silly games, multiplayer ‘live’ games that are updated frequently, and horror games are singled out as good content for streamers because of their unpredictability and potential for humorous or entertaining commentary, their encouragement of audience ‘back seat’ play, and their capacity for long-term play. By contrast, single-player narrative games, especially those with fairly linear stories, are seen as less amenable for streaming. This emerging discourse of streamability and discoverability contributes to a kind of normative standardization of which types of indie games and developers are considered commercially feasible, and which are not.

Many developers told us they take time to closely analyze the most popular games on Twitch and other platforms to determine what makes them so streamable, and whether those qualities are marketable to a wider audience beyond content creators. Hugh thinks the visual and user interface levels are crucial, to make the game legible and entertaining for audiences as well as players. His studio’s competitive multiplayer game was not made exclusively for streaming, but it was designed to work well as a competitive esport with online spectators. Its presentation is influenced by professional sporting events, ‘So we looked at both those types of, how those things are presented on TV and tried to copy certain things’. Hugh notes that designers may prefer simplicity and minimalism, but from a ‘spectator design’ standpoint it is important to have additional information visible on screen, such as timers and energy meters, to engage commentators and the audience in the action. In Helena’s experience some features streamers look for are relatively simple to implement, such as timers to foster speedrunning, but other features believed to enhance ‘streamability’, such as networked multiplayer, nonlinear structure, procedural generation to increase replayability, and customization, are more substantial undertakings for developers.

From Christopher’s perspective, every aspect of a game’s design is key to its appeal to streamers and viewers, and he put a lot of thought into making his multiplayer game ‘perfect for Twitch’. Having small teams, for example, allowed for legible communication and interaction between players without overwhelming the streamer or viewers (Christopher notes with pride that his team came to the same conclusion as popular AAA titles on the ideal team size). He also determined, based on observing streamers and the affordances of Twitch as a platform, that ‘games with some downtimes, as long as they’re not too long, is great because they have time to engage with their community and talk with people and read the chat’. This is somewhat counterintuitive, since Christopher’s design philosophy and past experience suggest players want a fast-paced game with as little downtime as possible. Tom also touches on this contrast: ‘the streamer demands a certain flow for it to fit inside of a stream. If I’m making a super high stressed action game, that doesn’t work for the streamer as well as it does for the individual player’. That ‘slow time’ allows streamers to more actively engage with their audiences, an essential part of their performance.

Developers are also keenly aware that if streamers are not hooked by a game’s pacing and flow early on, they may not stick with it for long. Curtis feels in retrospect that his most recent game was not structured well for streamers.

the big mistake that I didn’t know I was making until I saw it being streamed, which is that I was really trying to get a good difficulty curve from the game, which means sort of introducing things at a steady pace, but not necessarily showing our hand entirely early on. […] it’s only once you get into the second [world] that you start seeing the things that are important, that are not important, that are surprising and that make you sort of realize, “Oh, this game’s a lot deeper than I expected.” But that first world ends up being a really natural stopping spot. So, what I’ve seen is a whole bunch of people who’ve done a single stream of the game where they play for around half an hour to an hour, finish up the first line and then never come back to the game on stream because they feel this shows what the game is about.

This poses a dilemma, however, because Curtis feels the game as released is better from a design perspective, even if a more front-loaded structure would be more appealing for streamers and promotional purposes. As Christopher’s example of incorporating downtime also indicates, developers’ instincts about what works for ordinary players must be balanced against what they think will work for content creators, directly informing the design process.

#### Platform programmability and integrations

Twitch’s ‘programmability’ as a platform (Helmond, 2015) extends to game developers, who can use Twitch’s API (application programming interface) to easily integrate platform functionality directly into games – a more explicit way of enhancing streamability. The developers we spoke with are ambivalent toward these integrations. Melvin’s team added minor Twitch integrations that allow viewers to vote on in-game elements, which he says was a post-release decision once the game was already gaining popularity with streamers: ‘It was just a cool idea and there was a plugin that worked for it, so we used it’. Helena sees integrations as an iffy proposition that not all streamers actually like, especially if they are ‘obtrusive’ and allow viewers to directly intervene in the game, so her studio has stuck to ‘passive’ features like using viewer usernames for in-game characters. These kinds of features are fun add-ons rather than core to the game’s design. By contrast, Lauren has more experience with integrations and sees them as a substantial way to make genres perceived to be less streamable, such as single player narrative games, work well on stream. She explains that developers can tap into ‘that desire that streamers have to connect’ by developing features that allow streamers and viewers to engage directly through the game. One example is incorporating Twitch ‘drops’, free in-game items awarded to viewers if the streamer hits certain goals, which Lauren says incentivizes streamers to play the game, while simultaneously incentivizing viewers to become players so they can use the free items. But she cautions that it can’t be a tacked-on thing, adding ‘you have to actually think about it, I think developers are thinking about it more and more and are actually doing something that makes sense with their game, or just don’t do it’. Other developers are dubious of the value of integrations, especially for small teams on modest budgets, and Stuart notes that because they do not work on mobile devices, many viewers will not even be able to use them. Here we see a central, recurring tension between dedicating time, energy, and budget to make streaming an “integral feature” of the game, versus focusing on other things.

Several developers talked about plans to build future projects around streaming from the ground up. Holly is hoping to take advantage of the excitement around virtual reality (VR) systems, explaining a concept where ‘the streamer could play it in VR, but the audience could participate in the game itself using the new integration tools’, by voting on what happens in the game, with those interactions incorporated into the VR user interface so the streamer is not ‘cut off’ from the audience. An important factor for Holly is that these integrations are monetizable via viewer donation, with developers getting 20% of the revenue alongside the platform and the streamer, as opposed being left out of the deal as they are in other forms of streaming monetization. She sees this as a pathbreaking idea, since most VR games are not optimized or monetized for streaming, and hopes that the audience-interactive elements will also increase replayability. Curtis has also done experiments with what he calls ‘stream first’ games that are ‘made to be played over Twitch’. With some cultural agency funding, he prototyped ‘a game that was played between the audience and the person streaming’ using the Twitch chat, rather than the official API, and thought it was promising. However, he’s hesitant to turn it into a larger scale project due to the ‘serious money’ required and the lack of well-designed, successful examples of similar games, which he attributes to the fact that some audiences simply want to watch rather than become active participants in the game. Nevertheless, like most developers he has streaming front of mind as he conceptualizes new projects: ‘I’m going to just basically sit down and look at the state of the industry and try to figure out what my plans are next, because it keeps changing’. Indies are navigating a constantly shifting environment, and the language of risk permeates their comments.

For Tom, the greatest risk lies in ignoring streamers: ‘A lot of developers would make a game without considering necessarily whether they’re making it for streaming audiences’, waiting until the game is ready to release before contacting streamers with a ‘hope this works out’ approach rather than intentionality. Curtis finds this process ‘super annoying’, since he feels it devalues games designed to be self-contained experiences in favor of ‘endless amounts of content’ and games-as-a-service models. This is exacerbated by what he calls the inscrutable ‘black box of discoverability’ on different platforms, leaving developers mystified about how to find an audience. This skepticism is warranted, according to other developers. Hugh lists off the many ways incorporating streaming-related features can impact a project: ‘additional cost, additional programming time, additional quick fixing, additional [quality assurance]. So you have to be really sure that there’s value in what you’re doing before you commit to spend that money in development’. Christopher is fairly certain there is no value in streaming for his team’s next game, so he’s ‘not going to invest effort and money too much on streaming because these kinds of games almost [never] stream or barely’. Strategic decisions about costs and benefits, imagined audiences, and design ethos, all inflected by platform logics, are now central to commercial indie game development. These strategies are undertaken on the chance – however slim – that streaming can lead to commercial success or notoriety for indie developers.

### Streaming and indie game promotion

Although the experiences and specific attitudes of our interviewees vary, in the broadest terms indie developers see streaming as a means of promoting their games, alongside marketing, press, social media, public exhibitions, and other forms of promotion. According to developers, the potential value of streaming is highly dependent on the genre of game, and moreover there are many different kinds of streamers, each with different styles of performance and genre preferences, from competitive streamers who often play one game exclusively, to ‘variety streamers’ who rotate games and genres regularly, to ‘niche’ streamers who focus narrowly on a particular genre or subgenre. When the genre of game aligns with the streamers’ particular tastes or play style, Helena says, streamers become ‘very good hype people. If you have a game and you want people to get excited about it and you want to get it to as many people as possible, I feel like streamers are just the connectors’. Developers’ ground-up theories of streaming resonate with Austin Walker’s argument that the affordances of Twitch as a platform encourage a ‘promotional stance’ (Walker, 2014: 440). What exactly is being promoted – the game, the developer, the streamer, the platform, or some combination thereof – is not always apparent, however, which complicates notions of symbiosis between developers and streamers (Taylor, 2018: 126).

Some developers see a fairly direct connection between promotion, streaming, and sales. Melvin and Tessa’s accounts of the success of their ‘highly streamable’ competitive party game exemplify the idea of streamers as a form of promotion. Although they did see some spikes in their sales that directly correlated with popular streamers playing the game not long after its release, they place greater emphasis on the fact that they have maintained sales at an unusually steady level for upwards of 3 years, a ‘long tail’ of players discovering the game thanks in part to ongoing streaming and gameplay videos. ‘A lot of them are small, but still people are making content’, which for Melvin and Tessa speaks to the value of fostering paratextual practices as a ‘primary strategy’ for ongoing post-release promotion that they have pursued ‘pretty aggressively’ as they have pushed new content for the game by directly soliciting hundreds of individual streamers. Tessa puts it succinctly: streamers are ‘amplifiers’ and ‘arguably the most effective way that we could possibly have out there to get people’s attention and grow our audience’. Tom ascribes the modest popularity of his own humorous multiplayer game to its ‘replayability’, which he believes encouraged streamers, notably those who played in groups, to ‘keep coming back to it’, correlating with increased sales. Stuart had a similar experience when a high-profile YouTube content creator discovered one of his games several years after release, which he says ‘spiked my sales and then the sales reset, but not to launch, maybe to the year before. It basically, bumped it back a year in terms of the sales, in terms of those numbers. That’s huge’. Stuart directly attributes this ‘reset’ of his game’s long tail to this YouTuber, and he and Christopher both note that the permanent archive of recorded gameplay videos on YouTube may be an even greater asset than livestreamed content since they have more longevity. Several other interviewees drew similar correlations between streaming and long-term success, with the goal of becoming a ‘forever game’ updated over a long period of time for a dedicated audience, as Tom puts it.

Helena compares the role of streaming in promotion to celebrity and influencer marketing in other fields:

It’s why some perfume company would pay a model or a celebrity to take a picture with a perfume bottle. It’s like, we want the streamer to play the game because we know that will make the game seem fun to their audience.

While certainly this is true in the case of big-name celebrity streamers, smaller or niche streamers can also have a positive impact. Stuart says that his team is deliberately marketing their game to a particular genre niche: ‘our niche streamers are magnitudes smaller. But they are a way more targeted market. I feel like the conversion rate on views to sales would be way higher, like 10 times higher’ because they ‘cater directly to our audience’. In other words, quality is as important as quantity in promotion. Although he is not as convinced of direct sales boosts or measurable return on investment, Hugh contends that ‘we can definitely see that in some cases, our game brought audiences to a Twitch streamer’s channel. And in other cases, the Twitch streamers channel’s audience brought viewers for us for the game’. This leads him to contend that having a game streamed in sufficient numbers can improve discoverability on digital distribution platforms thanks to increased searches and wishlisting. In the same vein, Christopher sees streaming as a useful way to gradually build a player base for in-development games still in beta testing or early access.

### Uncertain results, ambivalence, and dismissal

In spite of the opportunities most of our interviewees see in streaming, the strongest theme in our conversations is ambivalence. Indies recognize the inevitability of streaming as a factor in contemporary game development, but frequently express uncertainty about how impactful, reliable, and measurable it really is, and whether actively pursuing it is worth the significant time and effort involved. Although as noted above some developers anecdotally attribute sales or engagement spikes to attention from specific streamers, in many other cases developers report that being featured by streamers with large followings produced no measurable results (Tran, 2020). Past success is no guarantee, either. When Tom made a new and improved 3D version of a previous game that had gained traction with many streamers, he found that they only played it briefly and moved on, and he isn’t sure why it didn’t resonate. Hugh characterizes indie game marketing as a process of ‘just testing assumptions constantly’, with no concrete rules or best practices to follow: ‘One week this type of content works, the next week this type of content works. You can’t plan for that. So I try a bunch of different things’. Helena likewise finds that there’s no formula, which makes it hard to track, lamenting that ‘the problem with streaming is that sometimes you can’t really judge if it’s working well’. This leads her to question whether exposure in and of itself is truly beneficial for her studio, contra popular narratives of streaming success.

In spite of his game’s popularity with streamers, Melvin also remains ambivalent. At one point, Melvin and Tessa’s studio invested money in the Twitch ‘Bounty Board’ system, which allows developers to make a pot of money available for streamers to claim in exchange for featuring their games. This led to more streamers playing the game, but didn’t have any obvious effect on sales or engagement. ‘What does that mean?’ Melvin wonders, frustrated,

Does that mean that it didn’t have any effect? Does it mean that the effect is going to be felt over the next 12 months as just like a long tail addition to the general visibility of the game? We don’t know.

Ultimately he concludes that Twitch is ‘trying to own the channel of communication between the developers and the streamer’, echoing Will Partin’s work on how platforms ‘capture’ previously off-platform monetization strategies (2020). Several other developers, including Tom, Helena, and Lauren likewise question the usefulness of paying streamers directly, at least for indies working with small budgets. Christopher’s studio used the Bounty system early on at Twitch’s urging and found that while it did get streamers to play the game, the return on investment in terms of sales was negligible, suggesting that, as Lauren puts it, the feature is ‘not attuned to indie reality’. Curtis links the pervasive uncertainty around streaming to the rapid pace of change in the game industry, and the dominance and inscrutability of platform algorithms: ‘Do articles make a difference? Do streams make a difference? Is there anything other than being on the front page of Steam, make a difference? And then no one knows how stuff gets onto the front page of Steam’. Lacking answers to these questions, Curtis concludes that all developers can do is find an intersecting point in the ‘Venn diagram’ of ‘games you want to make, games you can make, and games that have an audience’ and hope for the best. Other developers go so far as to chalk success with streamers up to sheer luck. Tom and Stuart both describe it as a ‘fluke’, with a high degree of uncertainty and unpredictability in terms of impact. Although streaming platforms, digital storefronts, and third-part analytics services offer developers a plethora of data about their games and players, these layers of quantification only seem to further mystify the process (Egliston, 2021).

All of this raises questions about much of the advice that circulates about streaming for indies. Charlie critiques the popular idea that if you ‘find the right streamer with the right audience […] it’s guaranteed to make all your financial dreams come true as an indie developer’ as a potentially dangerous misconception. Carolyn likewise observes that ‘people think it is an easier thing than it is’ and worries that naivety or overconfidence will lead developers to overemphasize streaming to the detriment other important factors. The concerns discussed above about how amenable different genres are for different kinds of streaming play into this as well. As Lauren puts it,

Considering streaming as just the one thing is kind of saying, all games are the same, all games have the same process […] can we realistically compare a three person VR studio to an 18 person mobile game studio? No, we can’t.

Lauren and Carolyn both caution that this makes it difficult to evaluate different indie experiences, since what works well for one game may not work at all for another.

For some indie developers, ambivalence leans toward a wholly negative view of streaming as too risky or even harmful to their games. This reflects a small but significant countercurrent to the generally celebratory discourse around game-based content creators, exemplified by Numinous Games’ charge that YouTube Let’s Play videos hurt sales of their narrative game *That Dragon, Cancer* (Green, 2016). Of all our participants, Holly’s perspective is the most negative and closely aligns with their experience:

more people have played the game for free than have bought it and I find that statistic depressing. […] it all comes back to the nature of our game. Our game is a narrative game that plays like a movie. Once you have seen our game, you don’t really have a reason to play it. And this is the inherent problem with the streaming culture and the game we made. The game we made, it streams well. People enjoy watching it and watching someone play it and it’s a cool experience, but they have no reason to buy it afterwards.

The issue was exacerbated by the fact that Holly’s team gave away numerous free promotional copies of the game to streamers, further reducing their overall sales. Anticipating these problems during development, her team considered asking streamers to only play half the game. They decided against it because they didn’t want to sour relationships by coming off as overly controlling, but their fears were borne out.

Another factor that contributed to Holly’s negative experience was her game’s serious, dark themes. She was worried that streamers – especially those who usually stream more mainstream games – would not take it seriously:

It feels dangerous to put it into the hands of someone who’s more likely to make fun of it than to appreciate it. […] I know there’s in theory no such thing as bad publicity, but since it’s such a specific and somewhat sensitive game, I just didn’t feel like we should be courting that kind of attention.

This again echoes Numinous Games’ concern that *That Dragon, Cancer*’s deeply personal story of loss would be devalued by content creators, and also resonates with the experiences of queer game developers like Robert Yang, whose games about gay sex and masculinity are frequent targets of gameplay reaction videos and streams that use them as fodder for exaggerated, often profane mockery, and have also been censored by Twitch (k, 2018; Yang, 2016). Helena, whose games often feature characters of diverse gender and sexual identity, is cautious about how sexist, homophobic, or racist ‘broey men’ streamers will present those aspects to their audiences. Increased visibility on the internet is not necessarily a positive thing, especially for people marginalized on the basis of identity, and game culture in particular is notoriously hostile (Gray, 2014; Nakamura, 2008).

All of this has left Holly fatigued by the overemphasis on streamers in indie game promotion, at least for narrative games: ‘frankly, I’m just disillusioned, I’m like why? Why would I do that? Cool, they’ll play it and no one will buy it’. Several other developers share this skepticism, with Tom even suggesting that a perceived decline in story-oriented games could be related to the rise of streaming, further evidence of a normative effect on game development.

### What exactly are streamers promoting?

A key factor in all of the different attitudes and perceptions discussed above is the knowledge that streamers are cultural producers in their own right. They may in some cases directly or indirectly promote indie games, but as noted above, cultural intermediation is not their primary function (Taylor, 2018: 51). This sets streamers apart from other actors in the space, such as journalists or festival curators, and developers are acutely aware of this fact. Tom observes that streamers cultivate ‘parasocial relationships’ that give their audiences a sense of a ‘personable and amicable’ social interaction when in fact it is largely unidirectional – concepts that align closely with critical research on other kinds of influencers (Abidin, 2015). Helena is also cautious about parasociality and worries about ‘the amount of trust that they get from their community, how easily influenced the community can be and rabid fans and the ways they can take advantage of that’. She points to controversies like *Counter-Strike: Global Offensive* YouTubers hawking gambling schemes as one example (Frank, 2017), and more recently there has been slew of sexual harassment and assault charges against popular streamers (Grayson, 2020). If a streamer recommends a game, that recommendation may hold additional weight thanks to their parasocial relationships (as in all influencer marketing), but streamers are less intermediating and more *remediating* the games they play – the stream stands as a distinct cultural product (Consalvo, 2017).

For some developers, this state of affairs feels unfair or even exploitative. Holly’s negative experience with her game has led her to personally view streamers as profiting off of indies: ‘If they have a large enough audience, they are literally getting money from the audience to be playing a game and or from I guess other ads on Twitch. […] They’re making money off of it’. On the other hand, Hugh understands why some developers feel this way but is critical of the impulse: ‘There is a particular angle that says streamers are parasites, they are producing content off the back of the work that we’re doing. […] the reality is that that’s just not how the world works anymore’. For Hugh, developers need to take streaming as a given of the contemporary industry and make the best of it, rather than treating streamers as competition. Similarly, Lauren argues that streamers and developers alike should approach streaming from a place of collaboration.

### Collaboration, connection, and community-building

It is in this potential for platform-mediated collaboration, connection, and community-building that developers see the most direct value in streaming. While the influence of streaming on direct or indirect sales is difficult to pin down, many interviewees point to other, less quantifiable but equally important factors at play, such as community-building and fostering audience engagement. What allows for long tail success like Melvin and Tessa’s is a critical mass of people invested in the game. Cultivating a loyal, participatory community of fan-consumers who feel a personal connection to the creator's work is understood to be essential for contemporary independent cultural production, and social media engagement is a key means of doing so (Baym, 2015; Kribs, 2017). In Carolyn’s experience, having your games featured on Twitch streams produces engagement ‘in a way that is very organic and/or authentic’, and so it should be seen as a community tool that ripples outward onto other social media platforms, regardless of sales. That sense of intimacy and authenticity is actively constructed and presents streamers as ‘real’ players actually playing and reacting to the game, often through ‘calibrated amateurism’ and other performative techniques, reinforced by the technical and social affordances of platforms (Abidin, 2018b; Cunningham and Craig, 2017; Ruberg and Lark, 2020). As Hugh argues, having an engaged community even if ‘they’re not all consumers or they’re not all potential purchasers of your product’ is useful in and of itself, giving developers more to work with as they relationally cultivate an audience, promote their games, and develop new projects.

Tessa, for example, uses streaming as raw material for producing social media posts for her studio: ‘for me it really serves the purpose of creating content that I can use to make the promotion of what’s coming up’. She collects clips of interesting or funny moments from streams, as well as memes, press, and other materials and reworks them into compelling content to share via other channels, a strategy other community managers also employ to generate engagement and build brand recognition. Tessa explicitly ties this to credibility and authenticity, a way of incorporating streamers and viewers into the studio’s community, and she says streamers appreciate this mutually beneficial acknowledgment. The community-building function extends also to shaping that community. Charlie argues that streamed and recorded play not only helps new players grasp the basics of a game, but additionally model normative ways of playing and enjoying it, contributing to emergent community standards more effectively than official developer-produced content or journalistic coverage. Although it was not a major theme in our interviews, some indie developers livestream their own game development work for similar pedagogical reasons (Consalvo and Phelps, 2021). Rather than seeing streamers as a way of outsourcing promotion, developers are compelled to adopt the same parasocial strategies of self-promotion and relational community maintenance as the streamers themselves, much like other independent cultural producers in the digital age (Kribs, 2017) – provided they have the time and resources to spend.

## Conclusion

Game developer perspectives on streaming illustrate just how mutable and precarious commercial indie game development continues to be, in spite of the proliferation of streaming-related success stories. The small Canadian developers we spoke with feel the influence of streaming on all aspects of their work and approach its potential risks and benefits ambivalently as they pursue the elusive goal of creative and economic sustainability (Whitson et al., 2018).

In the production process, streaming offers an opportunity for more organic playtesting and tweaking games in response to player experience, but this requires active, ongoing development work. Streaming also has a normative effect on design practices, as developers attempt to conceptualize games that appeal to streamers and viewers, though this may clash with their own design sensibilities. Programmable tools that integrate aspects of the streaming platform directly into games may enhance streamability, but they are often prohibitively costly or labor-intensive for smaller developers. Beyond production, streaming is understood to serve a promotional function, and some developers attribute sales bumps and long-term interest in their games to uptake by streamers. However, the majority of our participants express uncertainty about the value of streaming as a promotional tool, pointing to inconsistent results and frustratingly opaque platforms. For certain kinds of games, the impact of streaming is seen as largely negative, benefitting streamers and the platform more than developers, which has implications for what developers consider commercially feasible. Where developers seem to find streaming more consistently useful is in the less explicitly promotional but no less important community-building aspects of cultural production. Streaming thus becomes one of many venues where developers themselves are compelled to adopt the performative, relational techniques of streamers and other online influencers to cultivate a following for their work.

Our findings complicate the optimistic narratives and advice that characterize much of the discourse on streaming and indie games, in which platforms are paradoxically positioned as both the cause of and solution to the problem of discoverability. In fact, the ‘nested precarities’ of the competitive market for indie games, the rapidly changing game industry, and the ambiguous cultural and economic logics of different platforms (Duffy et al., forthcoming) are embodied in game developers as profound ambivalence (Chia, 2021). The experiences of indie game developers with livestreaming are thus consistent with the more general precarity and ambivalence of cultural work in the era of platform capitalism (de Peuter et al., 2017; Glatt and Banet-Weiser, 2021; Lehto, 2021; Siciliano, 2021). With a whole ecology of platforms and content creation practices shaping game production, promotion, monetization, and community management in the present moment, there is much to learn by centring the empirical experiences of ordinary game developers navigating this environment.
